# Supplementary material for: Reconciling Mining with the Conservation of Cave Biodiversity: A Quantitative Baseline to Help Establish Conservation Priorities
Source: PLoS One. 2016 Dec 20;11(12):e0168348. doi: 10.1371/journal.pone.0168348 (PMC5173368; doi:10.1371/journal.pone.0168348)
Supplement: S1 Dataset — (ZIP) [file pone.0168348.s002.zip › Taxa/Serra Norte/SN_2013/N5S/Lista N5S-25.pdf]

CAVIDADE N5S-0025

Classe	Ordem	Fam/Outros	Gên/Outros	Espécie	S-U	U-S	Zon.
Arachnida	Amblypygi	Charinidae	*Charinus*	sp.1	X	X	P
Arachnida	Amblypygi	Phrynidae	*Heterophrynus*	*longicornis*		1	P
Arachnida	Araneae	Ctenidae		jovem		1	P
Arachnida	Araneae	Ochyroceratidae	*Ochyrocera*	sp.1	X		P
Arachnida	Araneae	Ochyroceratidae	*Ochyrocera*	sp.3		X	P
Arachnida	Araneae	Ochyroceratidae	*Speocera*	sp.1	X		E
Arachnida	Araneae	Pholcidae	*Mesabolivar*	*eberhard*	X	X	E
Arachnida	Araneae	Theridiosomatidae	*Plato*	sp.1	X	X	P
Arachnida	Opiliones	Gonyleptidae		jovem	X		P
Arachnida	Pseudoscorpiones	Chthoniidae	*Pseudochthonius*	sp.2	X		P
Entognatha	Collembola	Entomobryidae		sp.1		X	P
Entognatha	Collembola	Isotomidae		sp.1	X		P
Entognatha	Collembola	Paronellidae		sp.4	X	X	E/P
Entognatha	Collembola	Tomoceridae		sp.1	X		E
Insecta	Coleoptera	Carabidae		sp.5	X	X	E
Insecta	Diptera	Psychodidae	*Sciopemyia*	*sordellii*		X	E/P
Insecta	Heteroptera	Lygaeidae	Rhyparochrominae	sp.1	X		E
Insecta	Heteroptera	Schizopteridae	Schizopterinae	jovem	X		P
Insecta	Heteroptera			sp.		X	P
Insecta	Homoptera	Cixiidae		sp.1	X	X	E/P
Insecta	Hymenoptera	Formicidae	*Crematogaster*	sp.1	X		E
Insecta	Hymenoptera	Formicidae	*Hypoponera*	sp.1	X	X	E/P
Insecta	Hymenoptera	Formicidae	*Pachycondyla*	*striata*	3	2	E/P
Insecta	Hymenoptera	Formicidae	*Pheidole*	sp.2	X		E
Insecta	Hymenoptera	Formicidae	*Rogeria*	sp.1	X		E/P
Insecta	Isoptera	Termitidae	*Nasutitermes*	sp.		X	P
Insecta	Lepidoptera	Noctuoidea		sp.4	X	X	E
Insecta	Orthoptera	Phalangopsidae	*Paraclodes*	sp.	2		E
Insecta	Orthoptera	Phalangopsidae	*Phalangopsis*	sp.	1	9	P
Insecta	Thysanoptera	Phlaeothripidae		sp.3	X		E
Gastropoda	Pulmonata	Systrophiidae	*Happia*	sp.	X		E
Malacostraca	Isopoda	Scleropactidae	*Circoniscus*	*buckupi*		X	P
Symphyla		Scutigerellidae	*Hanseniella*	sp.1	X	X	P
Amphibia	Anura	Strabomantidae	*Pristimantis*	cf. *fenestratus*	1		E
Mammalia	Chiroptera	Emballonuridae	*Peropteryx*	sp.		2	P
